# Interventional metabology: A review of bariatric arterial embolization and endovascular denervation for treating metabolic disorders

**DOI:** 10.1111/1753-0407.13437

**Published:** 2023-07-12

**Authors:** Zhi Wang, Dan‐Qi Zhu, Xiang‐Yun Zhu, De‐Chen Liu, Qing‐Yue Cao, Tao Pan, Qi Zhang, Xiao‐Chun Gu, Ling Li, Gao‐Jun Teng

**Affiliations:** ^1^ Center of Interventional Radiology and Vascular Surgery, Department of Radiology, Zhongda Hospital, School of Medicine Southeast University Nanjing China; ^2^ Department of Endocrinology, Zhongda Hospital, School of Medicine Southeast University Nanjing China; ^3^ Institute of Pancreas Southeast University Nanjing China

**Keywords:** intervention metabology, metabolic disorders, obesity, T2DM, 代谢干预, 代谢紊乱, 2型糖尿病, 肥胖

## Abstract

The rising prevalence of metabolic disorders such as obesity and type 2 diabetes mellitus (T2DM) poses a major challenge to global health. Existing therapeutic approaches have limitations, and there is a need for new, safe, and less invasive treatments. Interventional metabolic therapy is a new addition to the treatment arsenal for metabolic disorders. This review focuses on two interventional techniques: bariatric arterial embolization (BAE) and endovascular denervation (EDN). BAE involves embolizing specific arteries feeding ghrelin‐producing cells to suppress appetite and promote weight loss. EDN targets nerves that regulate metabolic organs to improve glycemic control in T2DM patients. We describe the current state of these techniques, their mechanisms of action, and the available safety and effectiveness data. We also propose a new territory called “Interventional Metabology” to encompass these and other interventional approaches to treating metabolic disorders.

## Graphical abstract

**Highlights**

- Interventional techniques offer novel therapeutic approaches for metabolic disorders such as obesity and type 2 diabetes mellitus, with advantages such as less invasiveness, fewer potential complications, and lower costs.
- By embolizing specific arteries feeding ghrelin‐producing cells, bariatric arterial embolization not only promotes weight loss but also has potential effects on glucometabolism.
- Endovascular denervation targets nerves that regulate metabolic organs to improve glycemic control. The detailed mechanism and long‐term effects are under research.
- A new territory named “Interventional Metabology” was proposed to encompass various interventional approaches to treating metabolic disorders.

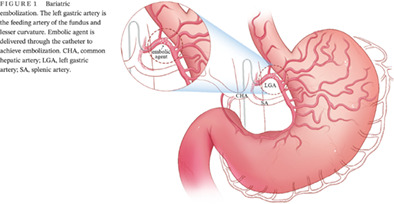

## 摘要

肥胖和2型糖尿病(T2DM)等代谢性疾病的患病率不断上升给全球健康带来了重大挑战。现有的治疗方法有局限性, 我们需要新的、安全的、创伤较小的治疗方法。代谢性疾病的介入治疗是一种新的治疗手段。本文主要对两种介入技术:减重动脉栓塞术(BAE)和血管内去神经术(EDN)进行综述。BAE通过栓塞特定的动脉造成胃饥饿素下降, 从而抑制食欲和促进体重减轻。EDN靶向调节代谢器官的神经, 改善T2DM患者的血糖控制。我们描述了这些技术的现状、作用机制以及可用的安全性和有效性数据。我们还提出了一个新的领域, 称为“介入性代谢学”, 以涵盖这些和其他介入性治疗代谢性疾病的方法。

## 1 INTRODUCTION

The prevalence of obesity and type 2 diabetes mellitus (T2DM) is increasing globally,^1^, ^2^ with the two conditions sharing essential pathophysiological mechanisms. Obesity is associated with insulin resistance and increased lipid metabolic abnormality, making it one of the major driving factors of the T2DM epidemic.^3^ T2DM is responsible for 90% of diabetes‐related mortality and disability worldwide.^2^ Although there are various therapeutic strategies for obesity and T2DM, including bariatric surgeries, pharmacotherapies, and lifestyle modification, their efficacy is limited in a number of patients.

Interventional radiology (IR) uses imaging modalities to guide minimally invasive procedures, thus reducing the complications associated with traditional surgery. IR has recently been introduced as a treatment for metabolic disorders, with bariatric arterial embolization (BAE) and endovascular denervation (EDN) emerging as two novel interventional procedures. This review aims to provide the latest evidence for these approaches.

## 2 BARIATRIC EMBOLIZATION

Behavioral modification, pharmacotherapy, and bariatric surgery are the main therapeutic approaches to obesity, with behavioral modification being the first‐line treatment,^4^ supplemented by pharmacotherapy.^5^ However, their long‐term efficacy is limited.^6^, ^7^ Although bariatric surgery is effective for obesity and has a high potentiality in T2DM treatment,^8^, ^9^ a number of individuals are ineligible because of their body mass index (BMI).^10^ Furthermore, its invasiveness may lead to intra‐ and postoperative problems.^11^ Other nonpharmaceutical therapies such as endoscopy and interventional bariatric and metabolic therapies offer more options to manage metabolic disorders.

**FIGURE 1:**
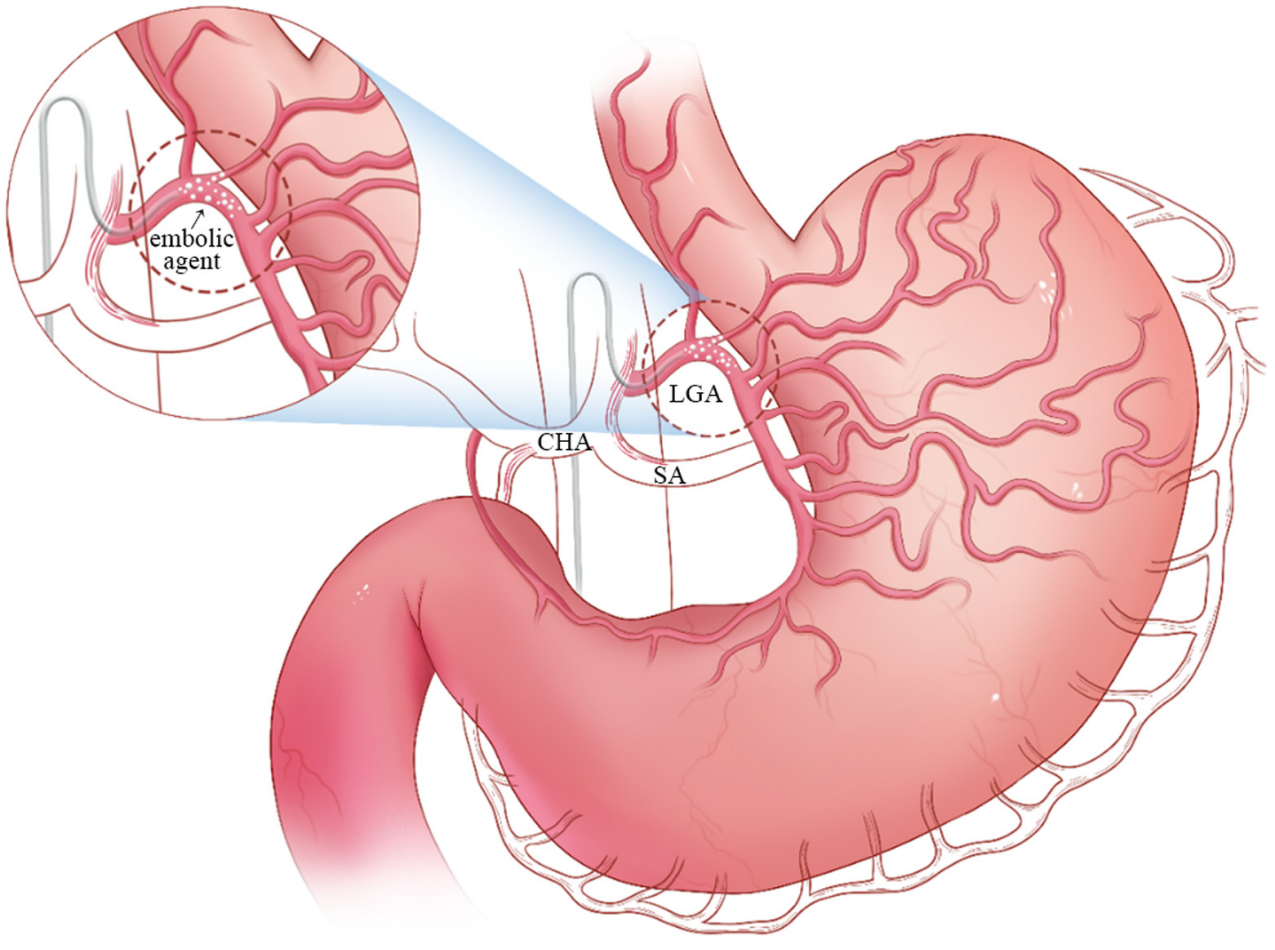
Bariatric embolization. The left gastric artery is the feeding artery of the fundus and lesser curvature. Embolic agents are delivered through the catheter to achieve embolization. CHA, common hepatic artery; LGA, left gastric artery; SA, splenic artery.

BAE is a novel treatment for obesity with the advantages of less invasiveness, fewer potential complications, and lower costs.^12^ This procedure includes embolization of the left gastric artery and/or other arteries supplying the fundus using embolic microspheres^13^, ^14^ (Figure 1). Currently, various animal experiments and clinical trials have been published to study the safety and efficacy of BAE in treating obesity. The rationale of BAE is to inhibit ghrelin, an orexigenic peptide hormone that increases food intake and promotes positive energy balance,^15^, ^16^, ^17^, ^18^, ^19^ by embolizing gastric arteries.^20^, ^21^, ^22^, ^23^ The predominance of ghrelin‐producing cells in the fundus makes BAE an effective treatment option.^24^, ^25^, ^26^, ^27^ BAE is well tolerated due to the abundant collateral circulation to the stomach, which prevents necrosis and perforation.

### 2.1 Preclinical and clinical evidence of BAE

BAE is effective in reducing weight and subcutaneous fat, inhibiting ghrelin production, and regulating various metabolic hormones in animal^14^, ^21^, ^23^, ^28^, ^29^, ^30^, ^31^ and human studies.^32^, ^33^, ^34^, ^35^, ^36^, ^37^, ^38^, ^39^ Preclinical studies using animal models have provided evidence of the feasibility and effectiveness of BAE (Table 1). In 2007, Arepally et al reported early experience using morrhuate sodium as an embolic agent in a swine model and showed that low and high doses can increase or decrease systemic ghrelin levels, respectively.^21^ Their further investigation demonstrated a short‐term persistence of ghrelin and weight gain reduction, with ulcers and gastritis verified by endoscopy.^20^, ^22^ Another study by Bawudun and colleagues using a canine model showed a substantial reduction in ghrelin, weight loss, and subcutaneous fat following chemical and polyvinyl alcohol particle embolization.^23^

**TABLE 1:** Preclinical studies of bariatric embolization for treating obesity.

Author (year)	Embolic	Animal model	Weight reduction	Ghrelin suppression	Ulceration rate	Other
Arepally et al (2007)^21^	Sodium morrhuate	Swine	Yes	a. Yes	NA	Ghrelin decreased with high dose and increased in low‐dose group
				b. No		
	a. high dose					
	b. low dose					
Arepally et al (2008)^22^	Sodium morrhuate	Swine	Yes	Yes	NA	NA
Bawudun et al (2012)^23^	a. bleomycin A5 hydrochloride‐lipiodol mixture	Canine	Yes	Yes	0/10	Subcutaneous fat size measured by computed tomography was significantly reduced
	b. microspheres (500–700 μm)					
Paxton et al (2013)^20^	Microspheres (40 μm)	swine	Yes	Yes	2/5	All ulcers occurred in nontarget locations.
Paxton et al (2014)^28^	Microspheres (40 μm)	swine	NA	NA	2/6	All treated animals showed minimal to mild sequelae of ischemia in the gastric fundus
Pasciak et al (2016)^30^	Yttrium‐90 microspheres	Swine	Yes	Yes	5/6	Three of five had healed superficial mucosal ulcers whereas the other two were predominantly healed with small central crater
Kim et al (2017)^29^	Microspheres	Swine	No	Yes	3/5	Recanalization was noted in three of five animals
	a. 150–250 μm (*n* = 3)					
	b. 50–150 μm (*n* = 2)					
Fu et al (2018)^31^	Microspheres	Swine	s			Plasma ghrelin was unchanged in both groups
	a. 100–300 μm		a. Yes	a. Yes	a. 5/5	
	b. 300–500 μm		b. No	b. No	b. 3/5	
Weiss et al (2020)^14^	Radiopaque microspheres (50 μm)	Swine	Yes	Yes	1/6	Calibrated Radiopaque microspheres and an antireflux catheter were used. Gastritis was absent in all animals

Several clinical trials have assessed the safety and effectiveness of BAE in humans (Table 2).^21^, ^33^, ^34^, ^35^, ^36^, ^37^, ^38^, ^39^, ^40^, ^41^, ^42^, ^43^, ^44^ The first‐in‐human BAE study was described by Kipshidze et al,^37^ which demonstrated that five patients had reduced appetite and weight after the procedure. Our team also reported 9‐month data in five patients and indicated that BAE is a safe and promising strategy for weight loss and abdominal fat reduction.^38^ Recently, the first randomized, sham‐controlled study of BAE demonstrated sustainable weight loss over a 12‐month follow‐up^43^ with substantial absolute and percentage total weight reduction as shown in the intention‐to‐treat and per‐protocol analyses. These results provided substantial evidence and support the clinical application of BAE to treat obesity.

**TABLE 2:** Clinical research of bariatric embolization for treating obesity.

Author (year)	Study design	Patients (n)	Body weight change	Body mass index change	Ghrelin suppression	Dietary consideration	Improvements in Glycemic control	AEs
Takahashi et al (2019)^40^	Retrospective	16	−6.4%	−6.3%	NA	NA	NA	NA
Kim et al (2018)^39^	Retrospective	21	−17.5%	−9.4%	NA	NA	NA	NA
Gunn and Oklu (2014)^41^	Retrospective	19 vs. 28	−7.3% vs. −2% (early)	NA	NA	NA	NA	NA
			−3.5% vs. −0.3% (late)					
Weiss et al (2017)^32^	Prospective, single‐arm	5	−12.8% (3 months)	NA	Yes	Yes	NA	2 minor AEs
Weiss et al (2019)^35^	Prospective, single‐arm	20	−8.2%	NA	NA	No	Yes	11 minor AEs
Zaitoun et al (2019)^33^	Prospective, single‐arm	10	−8.9%	−8.8%	NA	No	Yes	7 mild epigastric pain
Elens et al. (2019)^36^	Prospective, single‐arm	16	−10.0%	−11.8%	NA	No	NA	1 minor and 1 major AEs
Pirlet et al (2019)^42^	Prospective, single‐arm	7	−3.8%	NA	NA	No	NA	6 mild transient epigastric discomfort
Bai et al (2018)^38^	Prospective, single‐arm	5	−7.6%	NA	Yes	Yes	NA	No severe AE
Syed et al (2016)^34^	Prospective, single‐arm	4	−7.8%	NA	No	Yes	Yes	No major
								AE
Kipshidze et al (2015)^37^	Prospective, single‐arm	5	−17.2%	NA	Yes	NA	NA	NA
Reddy et al (2020)^43^	Randomized sham‐controlled	22 vs. 22	−6.4% vs. −2.8%	−6.7% vs. −2.7%	Yes	Yes	NA	1 serious AE unrelated to the procedure

### 2.2 Other metabolic effects of BAE

Weight loss improves glycemic control,^45^, ^46^ and several studies explored the metabolic effect of BAE. In a prospective study, BAE improved glycated hemoglobin (HbA1c) from 6% to 4.7% at 6 months post procedure in prediabetic patients.^33^ Meanwhile, Weiss et al^35^ reported a decrease in HbA1c at 12 months after BAE, although the improvements in glycemic control were independent of weight reduction. These findings revealed that BAE has the potential for glycemic control and that there may be other mechanisms apart from weight loss contributing to the improvements.

However, several concerns require resolution. First, although minor ulceration is a confirmed adverse event of BAE, whether it will cause functional changes such as gastric emptying and gastric acid secretion requires clarification. Second, although the underlying mechanism of BAE is currently considered to be ghrelin inhibition, the degree of weight reduction is not always correlated with ghrelin reduction. Finally, the potential role of BAE in glycemic control and its underlying mechanism requires further exploration. Recently, our team conducted a canine model experiment and found that, in addition to weight loss, which is one of the hypoglycemic mechanisms, BAE may contribute to delayed gastric emptying and therefore improve postprandial glucose excursion (unpublished). Despite this, further animal experiments and clinical trials are necessary before BAE becomes a widely used approach to treating morbid obesity and related metabolic disorders.

## 3 ENDOVASCULAR DENERVATION

The primary goal of treating T2DM is to control glycemia. Modulating the sympathetic nervous system (SNS), whose overactivation contributes to metabolic disorders, is a potential therapeutic strategy. Catheter‐based renal denervation (RDN) is a novel procedure that directly targets the SNS, but its efficacy in treating T2DM is inconsistent and not reproducible across studies. To target hepatic and islet innervation instead of renal nerves, we introduced denervation at the celiac and hepatic arteries, known as EDN, and explored the safety and efficacy in treating T2DM.^47^

### 3.1 The rationale for EDN

T2DM is often accompanied by SNS overactivation, exacerbating hyperglycemia and increasing the risk of complications.^48^, ^49^ The central nervous system regulates glucose metabolism through the direct innervation of organs by autonomic nerves. These nerves release neurotransmitters and neuropeptides to control hormone secretion, glucose synthesis, and metabolism.

**FIGURE 2:**
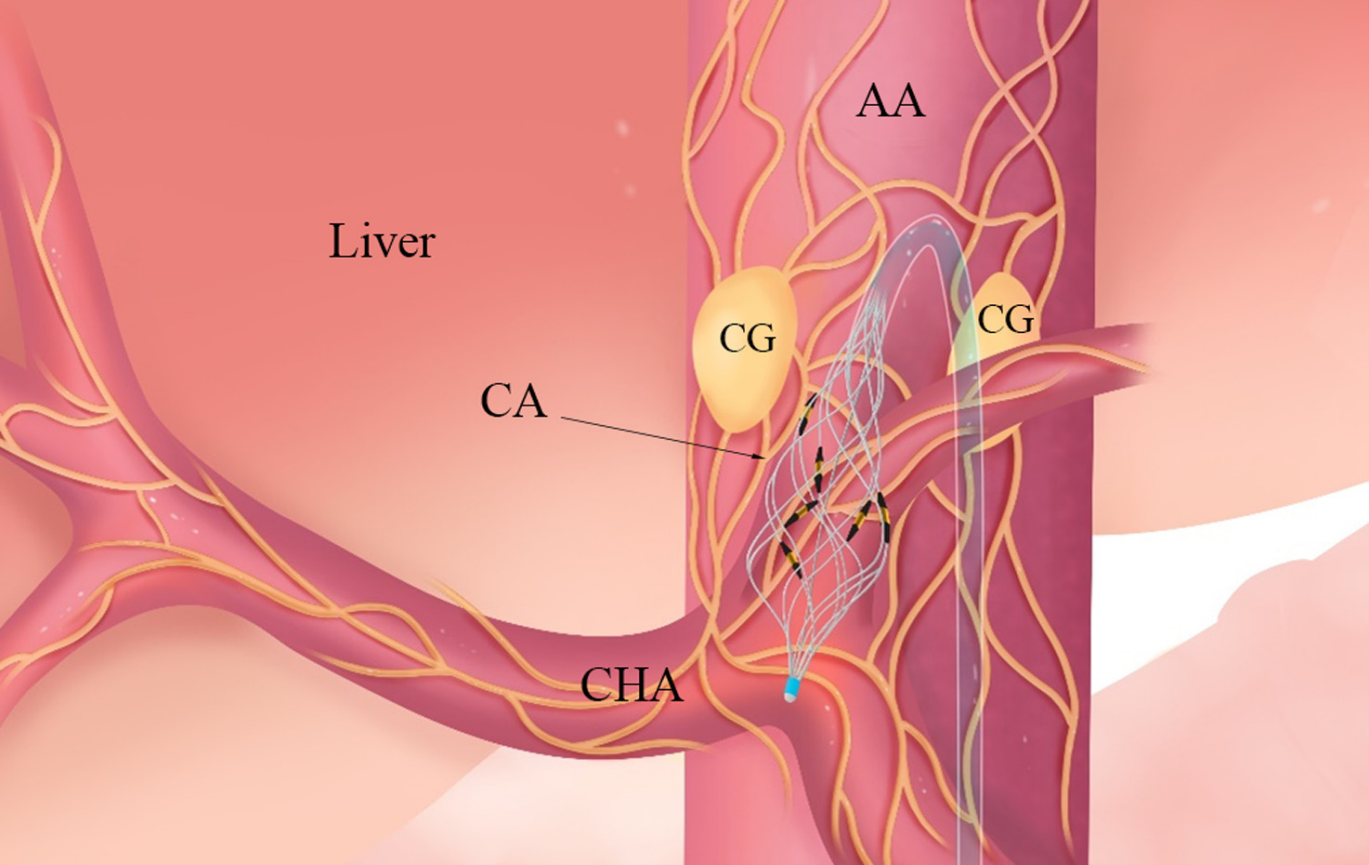
Endovascular denervation. The sympathetic nerves originating from the celiac ganglia run along the feeding arteries of the abdominal organs. The six‐electrode radiofrequency catheter is placed at the celiac artery to achieve denervation of the liver and islets. AA, abdominal artery; CA, celiac artery; CG, celiac ganglion; CHA, common hepatic artery.

Islets are densely innervated by sympathetic fibers that rapidly increase blood glucose levels by inhibiting glucose‐stimulated insulin secretion and stimulating glucagon secretion.^50^, ^51^ Meanwhile, the liver receives sympathetic innervation via the celiac and superior mesenteric ganglia^52^ (Figure 2). Hepatic sympathetic activity increases circulating glucose by promoting glycogenolysis and inhibiting glycogen synthesis.^53^ Consequently, the crosstalk between nerves and metabolic organs opens up an area for detailed studies on nerve modulation to regulate glucose metabolism and treat metabolic dysfunction.

RDN was first introduced to treat resistant hypertension and now enables selective denervation of the kidney. Although additional potential benefits such as metabolic improvements have been described in some small‐scale studies,^54^, ^55^ the outcomes vary across studies and are not always reproducible. For instance, the Denervation of the Renal Arteries in Metabolic Syndrome (DREAMS) study showed that RDN did not lead to a significant improvement in insulin sensitivity.^56^ A systematic review including 19 relevant studies suggested that although blood lipids were slightly improved, RDN appeared to have a negligible impact on metabolic parameters.^57^ Due to the lack of robust evidence that RDN could improve glycemic control, our team sought a new denervation site that could directly control metabolic organs such as the islet and liver.^47^ Neuromodulation of the sympathetic nerves of the islet and liver has demonstrated metabolic benefits, making denervation along the celiac and hepatic arteries a more reasonable option.

### 3.2 The preclinical evidence of hepatic denervation

Early research demonstrated that celiac ganglionectomy improved glucose tolerance in rats,^58^ and surgical or chemical sympathetic denervation of the common hepatic artery improves glucose tolerance and postprandial glucose clearance.^59^ Hurr et al^60^ showed that liver sympathetic nerve activity increased in the obese mice, and hepatic steatosis was alleviated by sympathetic denervation. Kraft et al^59^ established that surgical sympathetic denervation of the common hepatic artery in a canine model decreased both the hepatic and β‐cell defects caused by a high‐fat, high‐fructose diet, thereby increasing glucose tolerance. Kiuchi et al^61^ investigated the feasibility, safety, and efficacy of EDN of both hepatic and renal arteries in a swine model, achieving significant and sustained denervation without major safety events. Tzafriri et al^62^ analyzed the nerves and adjacent anatomies surrounding the human common hepatic artery to guide the clinical translation of catheter‐based denervation. They found that the hepatic artery is a rich and accessible target for sympathetic denervation. Recently, we used the high‐fat, high‐fructose canine model to study how EDN affects glucose metabolism. We found that EDN resulted in significant periarterial nerve injury and decreased tissue norepinephrine. Similar to hepatic surgical denervation, EDN led to a prominent increase of glucose tolerance by enhancing insulin secretion and improving insulin sensitivity of glucose disposal (unpublished). These results may provide further rationale for the clinical application of EDN.

### 3.3 The preliminary clinical evidence of EDN

Based on the preclinical evidence, we applied a minimally invasive, catheter‐based EDN procedure at the celiac artery and aorta around the celiac artery to treat T2DM.^47^ We registered the first‐in‐human study on ClinicalTrials.gov (NCT04086043) and reported the feasibility and safety of EDN in 11 patients with T2DM, as well as its initial efficacy. In our study, both HbA1c levels and homeostatic model assessment of insulin resistance were significantly reduced at 6 months (9.9% vs. 8.0%, *p* = .005; 13.3 vs. 6.0, *p* = .016). Additionally, we observed decreases in fasting plasma glucose and 2‐hour‐postprandial glucose levels (227.2 vs. 181.8 mg/dL, *p* < .001; 322.2 vs. 205.2 mg/dL, *p* = .001). Furthermore, EDN improved β‐cell function and led to a reduced insulin dosage. Surprisingly, we also observed an improvement in liver function (alanine transaminase, 31.0 vs. 24.0 U/L, *p* = .014; gamma‐glutamyl transferase, 47.0 vs. 27.0 U/L, *p* = .021). We did not find significant changes in blood lipids (triglyceride, 4.4 vs. 3.6 mmol/L, *p* = .244; total cholesterol, 5.1 vs. 4.6 mmol/L, *p* = .157). However, the metabolic benefits were not associated with changes in body weight, body mass index, or waist circumference (*p* > .05). Our study provides the first evidence of the clinical efficacy of EDN in treating T2DM. As a pilot study with a relatively simple design, we further initiated well‐designed studies to validate the efficacy and elucidate the clinical mechanism (NCT05631561, NCT05673668).

Before more applications of EDN, it is important to address several issues. First, the dose–response relationship requires clarification. Research has been conducted to study the optimal ablation parameters in RDN, and similar extensive data are needed for radiofrequency ablation in the celiac and hepatic arteries. Second, due to the comprehensive innervation of celiac sympathetic nerves, the effects on different organs need to be clearly understood to optimize ablation sites and reduce complications. Last, the long‐term effects of EDN have not yet been evaluated. Therefore, further preclinical experiments and clinical trials are still required to fully understand the potential risks and benefits of EDN.

## 4 CONCLUSION

IR offers a series of treatment options for metabolic disorders, leading us to propose a new territory named “interventional metabology.” The effect of BAE and EDN in combination with lifestyle intervention, pharmaceutical, and other treatments, as well as the comparison with standard care for obesity and T2DM are also arousing interest. Although reasonably safe, BAE and EDN require further investigation for unresolved issues and high‐class evidences.

## CONFLICT OF INTEREST STATEMENT

The authors declare that they have no competing interest.
